# Servant leadership and job satisfaction in medical assistants: mediating effect of work-life balance and burnout

**DOI:** 10.1108/JHOM-01-2026-0071

**Published:** 2026-07-14

**Authors:** Jayanthi Sebastian, John Harrison, Grace M. Hildenbrand, Shaoping Qiu

**Affiliations:** Department of Leadership Studies, Louisiana State University Shreveport, Shreveport, Louisiana, USA

**Keywords:** Medical assistants, Healthcare organizations, Employee well-being, Servant leadership theory, Work-life balance

## Abstract

**Purpose:**

The purpose of this research is to investigate the impact of servant leadership on the job satisfaction of medical assistants (MAs) in healthcare organizations across the United States of America, focusing primarily on the mediating effects of work-life balance (WLB) and burnout.

**Design/methodology/approach:**

This study adopted a quantitative, non-experimental, correlational and cross-sectional design. It utilizes Hayes's PROCESS model 4 with bootstrapping to test the hypotheses. Data were collected from 998 certified MAs in healthcare organizations in the United States of America using a purposive and convenience sampling method.

**Findings:**

The study finds that servant leadership positively predicts job satisfaction and WLB and negatively predicts burnout; servant leadership has a partial mediating effect on job satisfaction in the presence of both mediators.

**Research limitations/implications:**

The purposive and convenience sampling procedure decreased the generalizability of the findings. While survey of a broader sample of MAs was attempted, only a small percentage of the respondents were from healthcare organizations, and a majority of the participants were from the professional organization, the American Association of Medical Assistants. Using a quantitative approach did not allow for gathering information as in-depth as could be obtained with qualitative research. Although the study was open to all genders among the target population, the results were oriented towards the female population, since a majority of MAs were female.

**Practical implications:**

The findings from this study support that servant leadership influences the job satisfaction of MAs. Additionally, servant leadership can be utilized to improve compassion and understanding, increase communication, improve WLB and reduce burnout and maintain the well-being of MAs, which will significantly enhance the job satisfaction of the MAs. Furthermore, healthcare leaders and administrators may focus on creating a trusting relationship and empowering their MAs by displaying servant leadership characteristics. Overall, the research implications are that the perception of servant leadership positively impacts the job satisfaction of MAs.

**Social implications:**

The study validates the servant leadership theory, which operates primarily on empathy, in which the leader is a servant first and primarily focuses on the needs of the subordinates, which resonates with the MAs who put others first (Liden *et al.*, 2008). Next, the study validates the boundary theory with servant leadership supporting employees' well-being, with WLB as a mediator. The study also validates the job demands-resources model of burnout, demonstrating that burnout negatively impacts job satisfaction.

**Originality/value:**

The study examines whether servant leadership is a valuable leadership style positively impacting MAs' job satisfaction through improved WLB and reduced burnout. These findings support the theoretical frameworks applied and add to the existing literature of MAs, servant leadership and healthcare studies.

Medical assistants, allied healthcare workers who are the first point of contact with patients, play a crucial role in clinical operations and patient care across the United States. Lower levels of job satisfaction can affect the quality of patient care, and therefore, it is essential to understand the lack of job satisfaction among these healthcare employees.

## 1. Introduction

Medical assistants (MAs) are allied health personnel who work alongside physicians to provide basic patient care in outpatient clinics and perform administrative tasks such as scheduling patients (Gold and Harmes, 2022). For example, they provide patient care, such as obtaining patient histories, administering medication and preparing patients for diagnostic procedures. MA roles have expanded and transformed, and they contribute to the quality of care and team communication, assisting with documentation in the electronic health record, pre-visit planning, order entry and patient management (Gold and Harmes, 2022). MAs must work under the supervision of a registered nurse or physician, even though patients often confuse them with nurses.

Medical assisting is a fast-growing field expected to grow about 15% between 2023 and 2033 (Bureau of Labor Statistics, U.S. Department of Labor, 2024). According to the Health Resolution and Services Administration (HRSA) Health Workforce (2024), 90% of MAs are females. Women are often caregivers of aging parents, and their children are affected by work-life imbalance (Anuradha and Pandey, 2016). Also, employees often experience burnout and conflict in balancing personal and work obligations due to factors such as working long hours, information overload and time pressure, which can impact job satisfaction (Moric Milovanovic and Cvjetkovic, 2024). Moreover, the main factors that affect healthcare employees' job satisfaction are leadership style, work-life balance (WLB) and job burnout due to high workload and lack of autonomy (Fleuren *et al.*, 2024; Flores, 2021). Since highly satisfied employees deliver quality patient care, which enhances patient satisfaction (Chang *et al.*, 2017), it is imperative to examine the impact of leadership style on job satisfaction, WLB and burnout among MAs in the complex American healthcare system.

Servant leadership influences followers' job satisfaction (Barbuto and Wheeler, 2006; McCann *et al.*, 2014). Previous research has examined burnout and WLB under servant leadership in several disciplines around the world, including the relationship between servant leadership and job satisfaction among nurses and other healthcare professionals (Flores, 2021). Female nurses have been reported to have higher levels of stress compared to male nurses due to having multiple responsibilities, such as taking care of children and aged parents and working simultaneously, which is a lack of WLB (Satpathy *et al*., 2013). Similarly, Rotenstein *et al*. (2023) claim that burnout has been one of the main factors for job dissatisfaction; there had been complaints about it even before the COVID-19 pandemic among healthcare employees, such as nurses and physicians. To the best of our knowledge, however, little research has been conducted to examine servant leadership's impact on job satisfaction mediated by WLB and burnout among MAs in the United States.

The purpose of this study is to examine the relationship between servant leadership and its impact on MA job satisfaction in healthcare sectors in the United States. More precisely, this study aims to determine the mediating effects of WLB and burnout when considering the immediate manager's leadership style and the MA's job satisfaction.

## 2. Literature review and hypothesis development

MAs contribute to the quality of care and team communication, assisting with documentation in the electronic health record, pre-visit planning, order entry and patient management (Gold and Harmes, 2022). Lack of job satisfaction can affect the quality of patient care, which is an increased concern in the rapidly changing healthcare system in the USA (Chang *et al*., 2017). Furthermore, unsupportive leadership and organization, heavy workload, staff constraints, resistance to change, burnout and poor WLB among healthcare workers can affect job satisfaction (De Hert, 2020; Fernandes *et al.*, 2022).

The theoretical framework used to guide this study includes servant leadership theory (Greenleaf, 1977), boundary theory (Nippert-Eng, 1996), and the job demands-resources (JD-R) model of burnout (Demerouti *et al.*, 2001). Leadership begins within oneself, and people who care for others put others first, as in servant leadership, where the leader is a servant first (Greenleaf, 1977). Servant leadership theory promotes a holistic approach in which the primary goal of the leader is to serve others by prioritizing others (Greenleaf, 1977; Spears, 1996). A few significant characteristics of servant leadership are altruism, emotional healing and stewardship (Barbuto and Wheeler, 2006), which can be very well utilized in healthcare settings to create a positive impact on the employees to improve job satisfaction and WLB and to reduce burnout, especially among MAs.

Servant leadership behaviors emerged from Liden *et al*.'s (2008) instrument, with altruism as the central focus that fosters servant leadership's seven dimensions that will be surveyed in this study. Those dimensions include behaving ethically, conceptualizing, creating value for the community, emotional healing, empowering, helping followers grow and succeed and putting followers first (Liden *et al.*, 2014, 2015).

Boundary theory conceptualizes how employees create, maintain, alter, negotiate and preserve boundaries to organize and make sense of different life domains, particularly work and personal life (Nippert-Eng, 1996). The theory further posits that the separation between personal time and work exists along a continuum from segmentation to integration. While segmentation is a firm separation between work and non-work aspects, integration has less strong boundaries (Ashforth *et al.*, 2000; McCloskey and Crowne, 2023). Hence, understanding boundary theory to determine how MAs balance work-life is significant for the study to improve their job satisfaction.

The (JD-R) model of burnout proposes that working conditions can be categorized into two categories: job demands and job resources, which are distinctly related to specific outcomes since every job includes demands as well as resources (Demerouti *et al.*, 2001). Job demands are associated with the physiological and psychological costs and refer to the physical, social and organizational aspects of a job that require sustained mental and physical effort, which can lead to exhaustion, one of the leading causes of burnout (Maslach, 1998), from work. Meanwhile, job resources refer to physical, psychological, organizational or social aspects of the job that can be functional in reducing job demands at the physiological and psychological costs, achieving work goals and stimulating personal growth and development (Demerouti *et al.*, 2001). Thus, the JD-R model of burnout is a valuable framework that is utilized in the study to investigate burnout among MAs.

MAs can rely on clear role expectations to manage professional responsibilities alongside personal life with reference to boundary theory (Ashforth *et al*., 2000). Similarly, the JD-R model of burnout emphasizes that the high clinical and administrative demands faced by MAs require adequate resources, such as training and supervisory support, to reduce stress and prevent burnout (Demerouti *et al*., 2001). Together, these theories highlight that defined roles and sufficient organizational resources are essential for supporting the MAs' job satisfaction. Based on the theoretical framework, a conceptual framework was developed.

### 2.1 Servant leadership and job satisfaction

Job satisfaction is defined as any combination of physiological, psychological and environmental circumstances that causes a person to be satisfied or dissatisfied with their job, although there is no comprehensive definition to date (Hoppock, 1935). The relationship between intrinsic factors and extrinsic factors, such as exposure to incivility, which is more common among minorities and women, salary and rewards and the leadership style of the manager also decreases employees' job satisfaction (Cavanagh, 1992). Furthermore, job satisfaction is vital for a healthcare organization to pay attention to employees' job satisfaction during the early stages of the development of a quality system, since it is a primary component of the quality of human resources to achieve patient-centered care (Bhatnagar and Srivastava, 2012).

Servant leadership has demonstrated positive relationships with job satisfaction, work engagement, eudaimonic well-being of healthcare employees, patient satisfaction and hospital performance (Demeke *et al.*, 2024). Furthermore, healthcare organizations have been implementing changes in the leadership process by adopting the servant leadership style to achieve employees' job satisfaction and patient satisfaction since positive relationships from valuing people, sharing and providing exemplary leadership impact job satisfaction (Bennett and Hylton, 2020; Canavesi and Minelli, 2021; McCann *et al.*, 2014). Moreover, the leader's moral and ethical behaviors motivate the followers with trust and empowerment to be happy to work in a healthcare organization, demonstrating that servant leadership was positively related to employees' job satisfaction (Barbuto and Wheeler, 2006; Flores, 2021; Patrnchak, 2015; McCann *et al.*, 2014; Utama *et al.*, 2021). With reference to servant leadership theory, servant leader characteristics and behaviors (Greenleaf, 1977; Liden *et al*., 2014, 2015) demonstrate high-quality leader-employee relationships that positively relate to intrinsic and extrinsic components of job satisfaction. Based on the above literature, hypothesis H1 was proposed:

H1.
Servant leadership is positively related to medical assistants' job satisfaction.

### 2.2 Servant leadership and work-life balance

The relationship between servant leadership and WLB has received increased attention recently. Servant leaders can achieve the desired balance by understanding the employees' needs based on their psychological factors, although they can vary from person to person (Varma *et al.*, 2016). Moreover, servant leadership, as perceived by the employees, was negatively related to employees' emotional exhaustion and positively related to personal learning among 309 married employees, of whom 56% were females in a large commercial bank (Tang *et al*., 2015). WLB is the harmonious and holistic integration of work and non-work, which reflects a person's reaction to an unspecified level of balance and is generally thought to promote a person's well-being (Greenhaus *et al.*, 2003). Additionally, job and family demands decrease employees' WLB while job and family resources increase WLB (AlHazemi and Ali, 2016). WLB programs significantly improved nurses' well-being (Xiao *et al*., 2023). Boundary management preferences are generally predictors or moderators of work-family balance and work-family conflict (McCloskey and Crowne, 2023). It has been found that servant leadership has a positive effect on WLB and leaders' need to trust their followers, while WLB has a positive impact on behavior engagement and leaders' need to trust their subordinates among Indonesian healthcare employees (Tang *et al*., 2015). Based on the above literature, hypothesis H2 was proposed:

H2.
Servant leadership is positively related to medical assistants' work-life balance.

### 2.3 Work-life balance and job satisfaction

WLB is one of the core ingredients for job satisfaction and high-quality work performance (Susanto *et al.*, 2022). For the same reason, employees also need commitment to their jobs, specifically for patient satisfaction and fewer medical errors. Moreover, a healthy WLB helps individuals focus and maintain a high energy level and positive attitude. This combination fosters high productivity, which will likely help employees enhance their job performance, satisfaction and overall happiness (Abdullah *et al*., 2017). Apart from that, employees' job satisfaction increases when support from the managers and resources is high (Hngoi *et al.*, 2024). Further, decreased productivity, absenteeism, poor performance, high turnover and low job satisfaction were related to organizational outcomes, whereas lower family satisfaction, depression, headache, stress and anxiety were reported as family outcomes due to low job satisfaction (AlHazemi and Ali, 2016; Bella, 2023). Additionally, work-life conflict has been associated with psychological and physical health symptoms and medical errors in health care (Xiao *et al.*, 2023). Furthermore, job satisfaction will likely improve when employees are supported to balance their work and non-work activities (Abdullah *et al.*, 2017). Moreover, when inclusive policies are in place, effective WLB initiatives can lead to enhanced employee well-being and greater job satisfaction (Casper *et al.*, 2024). Based on the above literature, hypothesis H3 was proposed:

H3.
Work-life balance is positively related to job satisfaction among medical assistants.

### 2.4 Servant leadership, work-life balance and job satisfaction

WLB is likely an essential aspect for MAs to have high job satisfaction. Work-life imbalance and unsupportive leadership can reduce employee job satisfaction (Brough *et al*., 2020; Senol-Durak *et al*., 2021). Evidence suggests that WLB also mediates the association between job stress and mental health outcomes (Brough *et al.*, 2020; Timms *et al.*, 2015). Moreover, servant leadership has a positive effect on WLB and leaders' need to trust their followers, while WLB has a positive impact on behavior engagement and leaders' need to trust their subordinates among Indonesian healthcare employees (Setyaningrum *et al*., 2023). In summary, job satisfaction is the key for MAs to deliver high-quality patient care with empathy, which can be related to the leader's display of servant leadership and WLB. Based on the above literature, hypothesis H4 was proposed:

H4.
Work-life balance mediates the relationship between servant leadership and job satisfaction.

### 2.5 Servant leadership and burnout

Burnout is a specific kind of work-related strain resulting from chronic exposure to work-related stress (Lee *et al.*, 2011). If it is not successfully managed, it can contribute to reduced workplace efficacy, which has become one of the most significant occupational hazards, costing both organizations and individuals. Moreover, it has been linked to several physical and mental health problems, such as insomnia, anxiety, depression and gastrointestinal disturbances (Burke and Deszca, 1986; Lee and Ashforth, 1993). Empirical studies reported that servant leadership negatively impacts burnout, improves psychological health and the healthcare sector provides the most evidence of servant leadership's impact on burnout (Mahon, 2024). In addition, serving, empowerment, humility and authenticity can reduce workload strains, which can reduce burnout in healthcare organizations (Liden *et al.*, 2008, 2014, 2015; Winston and Fields, 2015). Servant leadership can add to positive outcomes of trust in supervisors, which also plays a key role in reducing burnout among healthcare employees (Babakus *et al.*, 2011). Hence, servant leadership can be utilized to address the working conditions: job demands and job resources, mentioned in the JD-R model of burnout. Based on the above literature, hypothesis H5 was proposed:

H5.
Servant leadership is negatively related to burnout among medical assistants.

### 2.6 Burnout and job satisfaction

Burnout, in general, negatively impacts job satisfaction since over-involvement in work and fewer entrepreneurial resources can cause erosion of job satisfaction due to burnout. Job satisfaction was inversely correlated with depersonalization and emotional exhaustion and positively correlated with personal accomplishment among physicians in Istanbul, suggesting that burnout is associated with decreased job performance and low career satisfaction (Ozyurt *et al.*, 2006). Also, unhappy employees tend to be dissatisfied with their jobs, and followers with a high degree of burnout report higher chronic work stress, stress-related illnesses and low job satisfaction (Mommersteeg *et al.*, 2006; Visser *et al.*, 2003). Employees with feelings of personal accomplishment, a core dimension of burnout, are happy and have high job satisfaction, which is associated with higher positive affect (Senol-Durak *et al.*, 2021). Furthermore, the well-being of an individual is referred to as having a lower negative and higher positive affect, lower levels of exhaustion and depersonalization, higher levels of personal accomplishments and better perceptions about life, which are also associated with job satisfaction (Randall and Scott, 1988). Additionally, Jachmann *et al.* (2025) found high levels of burnout among emergency healthcare workers, negatively affecting personal well-being and job satisfaction. Based on the above literature, hypothesis H6 was proposed:

H6.
Burnout is negatively related to job satisfaction among medical assistants.

### 2.7 Servant leadership, burnout and job satisfaction

High burnout and unsupportive leadership can reduce employee job satisfaction (Brough *et al.*, 2020; Obi *et al.*, 2020; Senol-Durak *et al.*, 2021). Also, working long hours and being over-involved can increase the levels of burnout among employees, such as MAs, which impacts their mood and can negatively affect job satisfaction and patient care (Brough *et al*., 2020; Senol-Durak *et al*., 2021). Additionally, empirical studies reported that servant leadership negatively impacts burnout, and the healthcare sector provides the most evidence of servant leadership's impact on burnout (Mahon, 2024). Similarly, Ahmed *et al.* (2025) show that servant leadership fosters psychological safety and resilience, conditions that are closely associated with higher job satisfaction and lower burnout. Overall, job satisfaction is the key for MAs to deliver patient care with empathy for higher patient satisfaction, and the impact of servant leadership on the job satisfaction of MAs in the United States will be determined using WLB and burnout as mediating variables. Based on the above literature, hypothesis H7 was proposed:

H7.
Burnout mediates the relationship between servant leadership and job satisfaction.

## 3. Conceptual framework

**Figure 1:**
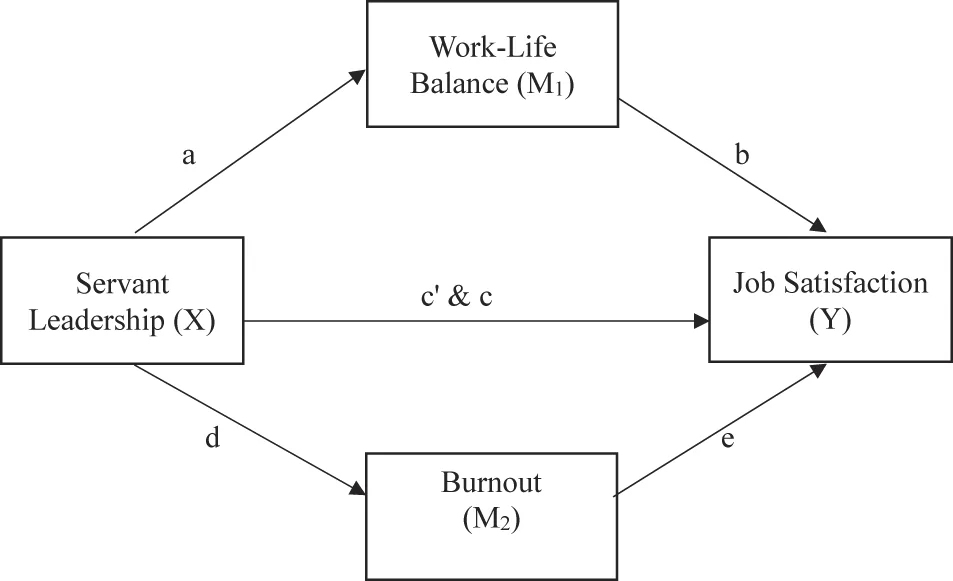
Representation of conceptual framework. Source: Authors' own work

In this study, WLB and burnout are used as mediating variables to examine the relationship between dimensions of servant leadership and job satisfaction. Job satisfaction is the dependent variable assessed in this study. A parallel mediation model analysis is used to examine the relationship between all the variables in the study. Hence, this study's conceptual framework is designed to investigate the relationship among servant leadership, WLB, burnout and job satisfaction of MAs in healthcare organizations in the United States of America (see Figure 1).

## 4. Methods

A quantitative, non-experimental and correlational design was utilized to examine the relationship between servant leadership, WLB, burnout and job satisfaction. After obtaining institutional review board approval from a university in the southern USA, authorization was obtained for the scales that needed permission. A survey link was then created on Qualtrics, and the link was sent to participants in healthcare organizations and the professional organization American Association of Medical Assistants (AAMA) national chapter, which sent an e-blast to their registered members and non-members. The state chapters of the AAMA were connected with the survey via Facebook. The study recruited participants nationwide, with a wide range of experiences from different specialties. The survey link directed participants to the screening and demographic questionnaires developed by the researcher, an informed consent form and the scales for all the variables in the study.

The target population for this study was certified MAs, recruited by a purposive and convenience sampling procedure. Based on the screening questionnaire, eligible participants had to work full-time with at least one year of experience in healthcare organizations in the United States and report directly to an immediate supervisor. Only the participants who said “yes” to all the questions in the screening questionnaire were qualified and were allowed to continue the survey. Then, they were requested to attest to the informed consent, and after attesting, they were shown the scales for the variables. The Bureau of Labor Statistics, U.S. Department of Labor, (2024) reports that there are about 763,000 MAs employed in the United States. Although The Research Advisors (2006) recommend a sample size of 384 participants with a 95% confidence level and a 5% margin of error, a sample size of 1,051 participants was collected.

The survey was kept open for four weeks in June 2025. Once the survey was completed, the data were extracted from Qualtrics and compiled in a Microsoft Excel document for the first round of data analysis. The collected data were visually analyzed for errors such as incorrect and incomplete entries and lines and duplicate IP addresses. Following the sanitization of the data set, it was uploaded into SPSS. The measures were added and checked for possible errors and incorrect entries. After cleaning all the errors, item two in WLB and items three and five in the short index of job satisfaction (SIJS) scales were reverse-coded. After sanitization of the data, there were a total of 1,004 respondents.

### 4.1 Measures

The servant leadership scale (SL-7) by Liden *et al.* (2015) was used to measure servant leadership. This scale has seven items; a sample item is “I would seek help from my leader if I had a personal problem.” Participants answered each item on a seven-point Likert scale that ranged from 1 (*strongly disagree*) to 7 (*strongly agree*). The Cronbach's alpha of the scale was 0.91 in the study.

The WLB Scale developed by Brough *et al*. (2014) was used to measure WLB. It is a four-item scale; a sample item is “I currently have a good balance between the time I spend at work and the time I have available for non-work activities.” Participants answer each item on a five-point Likert scale that ranges from 1 (*strongly disagree*) to 5 (*strongly agree*). The Cronbach's alpha of the WLB scale was 0.91 in the study.

The burnout measure shortform (BMS) by Malach-Pines (2005) was used to measure burnout. It has 10 items; a sample item of this scale is “Tired.” Participants answer each item on a 7-point Likert scale that ranges from 1 (*never*) to 7 (*always*). The Cronbach's alpha of the BMS scale was 0.92 in the study.

The SIJS by Judge *et al.* (2000) was used to measure job satisfaction. It has 5 items; a sample item of the scale is “I feel fairly satisfied with my present job.” Participants answer each item on a 5-point Likert scale that ranges from 1 (*strongly disagree*) to 5 (*strongly agree*). The Cronbach's alpha of the SIJS scale was 0.87 in the study.

## 5. Results

Following the data cleaning, the demographic information, assumption tests, descriptive statistics and hypothesis testing were performed, which are discussed in this section. Jamovi, statistical software, was adopted to determine the convergent and discriminant validity and the reliability of the variables in the study. Hayes's PROCESS Macro Model 4 with bootstrapping (Hayes, 2012) with 5,000 samples was also used in SPSS to test the hypotheses.

## 6. Demographics

Demographic information such as age, gender, race, marital status, highest level of education, state of residence, title of participants and the number of years worked as an MA was reported. A vast majority of the participants (97%) were female, which demonstrates the report from the Health Resources and Services Administration (HRSA) Health Workforce (2024) stating that 90% of MAs are female is reliable. This alignment suggests that the gender distribution observed in the study is closely reflected in national workforce trends. Furthermore, 30.7% of them were between the ages of 40 and 49, which represented the largest group of MAs. Also, 45.8% of participants had six to fifteen years of experience as an MA. Apart from that, 69.2% of participants had only an associate or technical degree. As for tenure, 46.0% of participants had six to fifteen years of experience as an MA. Based on the demographic information collected, 58.2% were married, and 80.7% were Caucasian or white.

### 6.1 Assumption tests

Participants in the study were sought using convenience sampling. Second, Likert scales were used to satisfy the interval or ratio scale assumption. Third, during the univariate outlier analysis using boxplots, six outliers were found in the dependent variable job satisfaction. Therefore, responses with the outliers were removed due to the availability of a large set of data, resulting in a total of 998 participants. Fourth, univariate normality was assumed based on the normal histograms. Furthermore, the values of skewness ranged from 0.31 to −0.51, and kurtosis ranged from −0.30 to −0.93. Fifth, Cook's Distance scores ranged from 0.00 to 0.03, and the assumption of no multivariate outliers was met. Sixth, the assumption of linearity was met when the scatter/dot matrix revealed an almost elliptical shape. Furthermore, the Pearson correlation coefficient ranged from −0.68 to 0.33 with a significance level *p* < 0.001. Seventh, the Durbin–Watson score was 2.05, which satisfied the assumption that the values of the residuals were independent. Eighth, the assumption of homoscedasticity was met since there was no obvious sign of funneling in the scatter plot. Ninth, the values of the residuals were normally distributed since the dots that represent the residuals were close to the diagonal on the P-P plot. Finally, the assumption of multicollinearity, which is one of the most crucial assumption tests, was met since variance inflation factor ranged from 1.30 to 1.68 and tolerance scores ranged from 0.60 to 0.77. Furthermore, the results from Harman's one-factor test showed that a single factor explained 41.2% of the total variance, indicating common method variance is unlikely to be a serious concern. Thus, the data met all the assumptions and were ready for hypothesis testing.

### 6.2 Model fit

Factor model fit (see Table 1) was acceptable: *χ*^*2*^ (294) = 1,556, *p* < 0.001, *root mean square error of approximation* = 0.06,

**Table 1:** Fit indices for the model

Model	*χ* ^*2*^ *(df)*	*RMSEA*	*CFI*	*TLI*	*SRMR*	*p*
Mediation model	1,556 (294)	0.06	0.95	0.94	0.09	<0.001

**Note(s):** *N* = 998

**Source(s):** Authors' own work

*Comparative fit index* = 0.95, *Tucker–Lewis index* = 0.94, *standardized root mean square residual (SRMR)* = 0.09. The fit indices indicate a slightly less than optimal fit since SRMR is above the threshold of 0.08. However, most fit indices are good, demonstrating an acceptable model fit.

### 6.3 Reliability, convergent validity and discriminant validity

Table 2 presents the average variance extract (AVE), in which all AVE values are moderately larger than 0.50, demonstrating good convergent validity. The square root of AVE for SL7 = 0.78, WLB = 0.86, BMS = 0.82 and JS (Job Satisfaction) = 0.76 revealed that the square root of AVE for all factors exceeded the intercorrelations as shown in Table 3, indicating good discriminant validity. For reliability, the Cronbach's alpha of servant leadership was 0.91, WLB was 0.92, burnout was 0.92 and job satisfaction was 0.87 (see Table 2). All values exceeded the suggested threshold of 0.7, demonstrating a higher level of reliability. Thus, the measures demonstrated good reliability and validity.

**Table 2:** Reliability indices

Variable	α	AVE	Sqrt.AVE
Servant leadership	0.91	0.61	0.78
Work-life balance	0.92	0.74	0.86
Burnout	0.92	0.67	0.82
Job satisfaction	0.87	0.58	0.76

**Note(s):** *N* = 998, *p* < 0.001

**Source(s):** Authors' own work

**Table 3:** The square root of AVE and the inter-factors correlation

	Servant leadership	Work-life balance	Burnout	Job satisfaction
Servant leadership	0.78			
Work-life balance	0.33*	0.86		
Burnout	−0.48*	−0.56*	0.81	
Job satisfaction	0.56*	0.46*	−0.68*	0.76

**Note(s):** *N* = 998, *p < 0.001; *M* (Median), *SD* (Standard Deviation)

**Source(s):** Authors' own work

### 6.4 Descriptive statistics and intercorrelations

The descriptive statistics (see Table 4) showed that the mean for all variables ranged from 3.29 to 4.58, and the standard deviation ranged from 0.86 to 1.52. The correlations between the variables were between +1 and −1 and did not exceed 0.80, with the strongest being −0.68 and the weakest being 0.33, while the correlation coefficient (*r*) was −0.48 between burnout and servant leadership and 0.33 between WLB and servant leadership.

**Table 4:** Descriptive statistics and correlation coefficients

	*M*	*SD*	Servant leadership	Work-life balance	Burnout
Servant leadership	4.58	1.52			
Work-life balance	3.29	1.06	0.33*		
Burnout	3.38	1.26	−0.48*	−0.56*	
Job satisfaction	3.53	0.86	0.56*	0.46*	−0.68*

**Note(s):** *N* = 998, *p < 0.001

**Source(s):** Authors' own work

### 6.5 Hypotheses testing

**Figure 2:**
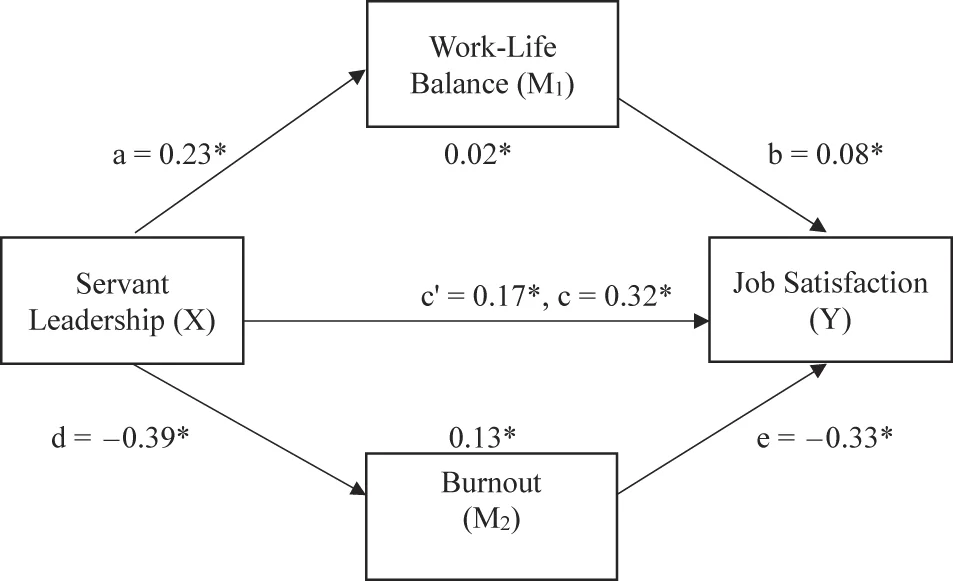
Path diagram for mediation model. Source: Authors' own work

The results showed that servant leadership has a significant impact on MAs' job satisfaction, supporting H1, the total effect (c), (*b* = 0.32, *t* = 21.33, *p* < 0.001, *LLCI *(Lower Level Confidence Interval)= 0.29 and *ULCI *(Upper Level Confidence Interval) = 0.35). Additionally, the results showed that servant leadership has a significant impact on WLB, supporting H2, which is path a (*b* = 0.23, *t* = 11.05, *p* < 0.001, *LLCI* = 0.19 and *ULCI* = 0.27). The results also showed that WLB has a significant impact on the dependent variable job satisfaction, supporting H3, which is path b, (*b* = 0.08, *t* = 3.63, *p* < 0.001, *LLCI* = 0.04 and *ULCI* = 0.12). Furthermore, H4 was tested to examine if WLB mediates the relationship between servant leadership and job satisfaction, and the results showed that the indirect effect of WLB was statistically significant (*Effect* = 0.02, *BootSE* = 0.01, *BootLLCI* = 0.01 and *BootULCI* = 0.03), supporting H4. Also, the results showed that servant leadership has a significant impact on burnout, which is path d, supporting H5, burnout (*b* = −0.39, *t* = −17.05, *p* < 0.001, *LLCI* = −0.44 and *ULCI* = −0.35). Additionally, the results showed that burnout has a significant impact on the dependent variable job satisfaction, path e, supporting H6 (*b* = −0.33, *t* = −17.57, *p* < 0.001, *LLCI* = −0.37 and *ULCI* = −0.30). Finally, the results showed that the indirect effect of burnout was statistically significant, with no zero between the bootstrap lower-level confidence interval and the bootstrap upper-level confidence interval, supporting H7 (*Effect* = 0.13, *BootSE* = 0.01, *BootLLCI* = 0.11 and *BootULCI* = 0.15). Thus, all hypotheses tested were significant (see Table 5). Furthermore, the mediation effects of WLB and burnout demonstrated a partial mediation of servant leadership on the job satisfaction of MAs (see Figure 2).

**Table 5:** Hypotheses results

		Effect	Se	BootSE	*t*	*p*	LLCI	ULCI	BootLLCI	BootULCI
H1	Path c	0.32	0.01		21.33	<0.001	0.29	0.35		
H2	Path a	0.23	0.02		11.05	<0.001	0.19	0.27		
H3	Path b	0.08	0.02		3.63	<0.001	0.04	0.12		
H4	WLB	0.02		0.01					0.01	0.03
H5	Path d	−0.39	0.02		−17.05	<0.001	−0.44	−0.35		
H6	Path e	−0.33	0.02		−17.57	<0.001	−0.37	−0.30		
H7	B	0.13		0.01					0.11	0.15

**Note(s):** Independent variable: SL (Servant Leadership), M_1_: WLB (Work-Life Balance), M_2_: B (Burnout), dependent variable: JS (Job Satisfaction), *N* = 998

**Source(s):** Authors' own work

## 7. Discussion

This study examined the mediating role of WLB and burnout on the relationship between the perceived servant leadership of immediate managers and job satisfaction among MAs. The results showed that servant leadership had a significant effect on job satisfaction. The results also demonstrated that the mediating variables WLB and burnout had a significant effect on the job satisfaction among MAs, and therefore, the study determined that servant leadership had a partial mediating effect on the job satisfaction of MAs in the United States.

The study indicated that there is a positive correlation between the perception of servant leadership and MA job satisfaction. This finding is consistent with the existing literature that servant leadership is an excellent approach in healthcare settings for job satisfaction (Barbuto and Wheeler, 2006; Liden *et al.*, 2014, 2015). The results also showed that servant leadership has a significant impact on WLB, which is aligned with the previous studies that have found a positive impact on the WLB of employees (Lamprinou *et al.*, 2021; Setyaningrum *et al.*, 2023). WLB has a positive impact on job satisfaction (Abdullah *et al.*, 2017; Anuradha and Pandey, 2016). There was a significant negative effect of servant leadership on burnout, supporting the existing literature that servant leadership can reduce the burnout of employees by prioritizing the employees' well-being (Babakus *et al.*, 2011; Liden *et al.*, 2008, 2014, 2015). Burnout has a significant negative impact on job satisfaction, which is supported by previous studies (Ozyurt *et al.*, 2006; Randall and Scott, 1988). Furthermore, burnout mediated the relationship between servant leadership and job satisfaction, which aligns with the existing literature that indicates that managers can reduce burnout and improve job satisfaction (Randall and Scott, 1988; Tahar *et al.*, 2022).

The research findings supported all the hypotheses. Additionally, this outcome not only reinforces the initial assumptions but also contributes uniquely to the body of research on MAs. The results provide new insights and add value to existing literature, highlighting the importance and relevance of the research in advancing the understanding of MAs' roles and effectiveness through the variables used in this study.

### 7.1 Limitations

There are several limitations in this study. First, the purposive and convenience sampling procedure decreases the generalizability of the findings. Second, the study attempted to survey a broader sample of MAs but was able to obtain only a small percentage of the respondents from healthcare organizations, and a majority of the participants were from the AAMA. Third, using a quantitative approach did not allow for gathering information as in-depth as could be obtained with qualitative research. Fourth, the study being correlational, causation could not be established for the model. Fifth, although the study was open to all genders among the target population, the results were oriented towards the female population, since a majority of MAs were female.

### 7.2 Recommendations for future research

A qualitative or mixed-method study design can also be recommended, since it will help to sense emotions and the personal experience of MAs for a deeper understanding. Studies based on work experience and age might also be beneficial to understand WLB, burnout and job satisfaction, since experience might impact these factors. To add to the theory of servant leadership, future studies must examine the role of other mediators, such as resilience, employee engagement and increased work experience, on the relationship between servant leadership and job satisfaction among MAs. Since servant leaders can create a positive work environment and reduce employees' absenteeism and turnover, servant leadership can also be explored in terms of turnover. Overall, more research is needed on servant leadership, WLB, burnout and job satisfaction to understand these factors in healthcare settings.

## 8. Implications

The study offers valuable information for theory and research in servant leadership and job satisfaction, and it examines two other important aspects, namely WLB and job satisfaction, which are critical factors that can affect MAs' job satisfaction. Therefore, this study has significant theoretical and practical implications.

### 8.1 Theoretical implications

The study validates the servant leadership theory, which operates primarily on empathy, in which the leader is a servant first and largely focuses on the needs of the subordinates, which resonates with the MAs who put others first (Liden *et al.*, 2008). Next, the study validates the boundary theory with servant leadership supporting employees' well-being, with WLB as a mediator. The study also validates the JD-R model of burnout, demonstrating that burnout negatively impacts job satisfaction.

### 8.2 Practical implications

The findings from this study support that servant leadership influences the job satisfaction of MAs. Healthcare organizations can improve MAs' job satisfaction by implementing leadership training that emphasizes servant leadership behaviors in everyday supervisory practice. From a boundary theory perspective, training leaders to respect non-work boundaries and support boundary clarity may help MAs better manage work and personal demands. Consistent with the JD-R model, servant leadership training can equip supervisors to enhance job resources, such as emotional support, empowerment and open communication, while actively reducing excessive job demands that contribute to burnout. By embedding servant leadership, boundary awareness and resource-building strategies into leadership development programs, healthcare organizations may promote employee well-being, reduce burnout and support sustainable job satisfaction among MAs. Overall, the research implications are that the perception of servant leadership positively impacts the job satisfaction of MAs.

## 9. Conclusion

This research offers important insights and information on the complex interactions between servant leadership, WLB, burnout and job satisfaction among MAs. The study indicates that there is a significant positive impact of servant leadership and WLB on job satisfaction. The study also indicates that there is a significant negative impact of servant leadership on burnout, and burnout has a negative effect on job satisfaction. Both mediating variables significantly mediated the relationship between servant leadership and job satisfaction. The study's findings contribute to the body of knowledge on how servant leadership in healthcare can influence MAs' job satisfaction with the mediating effects of WLB and burnout for high-quality patient care. Overall, servant leadership has a partially mediated effect on job satisfaction.
